# Determination of the Minimal Clinically Important Difference (MCID) for Ocular Subjective Responses

**DOI:** 10.1167/tvst.13.8.28

**Published:** 2024-08-16

**Authors:** Maria Navascues-Cornago, Sarah Guthrie, Philip B. Morgan, Jill Woods

**Affiliations:** 1 Eurolens Research, Division of Pharmacy and Optometry, Faculty of Biology, Medicine and Health, The University of Manchester, Manchester, UK; 2 Centre for Ocular Research & Education (CORE), School of Optometry & Vision Science, University of Waterloo, Waterloo, ON, Canada

**Keywords:** minimal clinically important difference, visual analogue scale, contact lenses, ocular comfort/dryness, patient-reported outcomes

## Abstract

**Purpose:**

To determine the minimal clinically important difference (MCID) for contact lens (CL)–related subjective responses and explore whether MCID values differ between subjective responses and study designs.

**Methods:**

This was a retrospective analysis of data from seven one-week bilateral crossover studies and 14 one-day contralateral CL studies. For comfort, dryness, vision, or ease of insertion, participants rated on a 0–100 visual analogue scale (VAS) and indicated lens preference on a five-point Likert scale featuring strong, slight, and no preferences. For each criterion, four MCID estimates were calculated and averaged: mean VAS score difference for “slight preference,” lower limit of 95% confidence interval VAS score difference for “slight preference,” difference in mean VAS score difference between “slight” and “no preference” and 0.5 standard deviation of VAS scores.

**Results:**

The four calculation methods generated a small range of MCID values. For bilateral studies, the averaged MCID was 7.2 (range 5.4–8.8) for comfort, 8.1 (5.2–10.6) for dryness, 7.1 (5.5–9.3) for vision and 7.6 (6.0–10.5) for ease of insertion. For contralateral studies, the averaged MCID was 6.9 (6.1–7.6) for comfort at insertion and 7.5 (6.8–8.2) for end-of-day comfort.

**Conclusions:**

This work demonstrated very similar MCID values across subjective responses and study designs, in a population of habitual soft CL wearers. In all cases, MCID values were on average seven units on a 0 to 100 VAS.

**Translational Relevance:**

This work provides MCID values which are important for interpreting ocular subjective responses and planning clinical studies.

## Introduction

**Figure 1.**
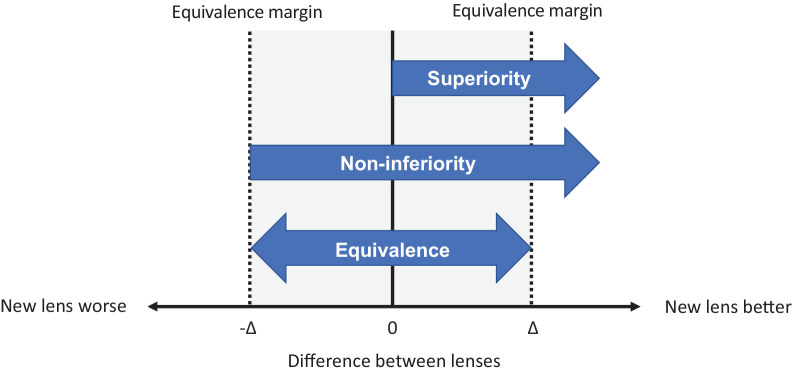
Superiority, non-inferiority, and equivalence testing in clinical trials. Δ: margin for equivalence/non-inferiority.

The objective of many clinical trials is to compare two or more products, and this extends to the medical devices industry. In the contact lens field, it has been commonplace to design “superiority” studies, seeking to show improved performance with one contact lens type over another. In the 1990s and 2000s, when there were major advances in materials and manufacturing technology, differences were relatively easy to confirm with statistical tests to establish statistical *superiority* (i.e., when the lower 95% confidence limit of the differences between products for specific performance attributes is greater than zero) (Fig. 1).^1^^,^^2^ Advancements in contact lens material, design, and manufacturing over the past few decades have led to significant improvements in contact lens products, and contemporary contact lenses have reached a level of clinical performance that it has become increasingly difficult to achieve a major breakthrough in product performance. As a result, it can be challenging to assess clinically meaningful differences in the performance attributes of commercially available contact lenses.

The typical elements of soft contact lens performance assessed in clinical trials are visual acuity, lens fit, ocular surface physiology, and subjective comfort. Typically, commercial contact lenses today provide excellent comfort and vision, optimal fit and have little impact on physiology.^3^ To identify improved products which will deliver better outcomes for wearers, current clinical trials increasingly relying on subjective ratings to differentiate products, using tools such as visual analogue scales (VAS)^4^^–^^7^ and Likert scales.^8^^,^^9^ These scales are now commonly used to capture the subjective elements of lens performance including comfort, vision and aspects of lens handling. However, as soft lens materials have become more advanced, even these subjective measures of contact lens performance have become similar between lenses and proving statistical superiority has become challenging.^10^ On the other hand, with very large samples or statistical power, small differences can turn out to be statistically significant yet not clinically relevant.

Because of the challenges of establishing superiority to already excellent contact lens products, the more modest goal of establishing either equivalence or non-inferiority have become more commonly used to assess new contact lens products.^11^^,^^12^ Equivalence trials are set up to statistically demonstrate that the difference between two products with respect to an outcome parameter falls within a pre-specified range of values, as defined by an “equivalence margin,” a value that is clinically meaningful (Fig. 1). The aim of *non-inferiority* trials is to statistically demonstrate that a new product is not worse than an existing product by more than this pre-specified margin (Fig. 1). The knowledge that a contact lens product is *equivalent* or *non-inferior* to a well-established existing product for certain performance indicators can be very useful for eye care professionals (ECP). For example, an ECP might select a new product which is equivalent in terms of fit, vision and comfort to an existing product but which has secondary attributes such as a greater number of stock-keeping units or a lower price.

Numerical rating scales or VAS are often used to assess the subjective performance of contact lenses.^4^^–^^7^ When interpreting outcomes from these scales, it is important to determine whether a difference in scores between two different products is really indicating a difference in performance (i.e., a meaningful difference) or just a limitation of how accurate humans are when converting a subjective feeling or experience into a numeric value. Additionally, contact lens wearers may be able to detect a difference between two products and subsequently assign different scores. However, this difference in score may be perceived by the wearer as relatively insignificant or having little importance. Therefore, in addition to using statistical criteria to evaluate subjective scores in clinical studies, there is a need to evaluate these scores based on their relevance from the wearer's point of view.

To interpret the clinical relevance of patient-reported outcome measures (PROMs), it is essential to establish the minimal clinically important difference (MCID). The MCID has been defined as “the smallest difference in score in the domain of interest which patients perceive as beneficial and which would mandate, in the absence of troublesome side effects and excessive cost, a change in the patient's management.”^13^^,^^14^ In a contact lens context, MCID is the score at which patients report a noticeable difference in comfort, vision, or some other measure. For example, Papas et al.^5^ reported that for rating soft lens comfort, a difference of about 7 to 8 units on a 0–100 scale can be regarded as the just-noticeable difference (JND), which indicates the lowest change or difference likely to be clinically significant. This JND value was determined by assessing interocular preference in subjects wearing identical pairs of contact lenses for 15-minute periods. It is not known whether this finding can be extrapolated to assessments of bilateral lens wear or contact lens use of longer duration. Once determined, the MCID for a scale can then be used to plan meaningful clinical studies as the MCID can be used as the *equivalence or non-inferiority margin* in statistical testing. In addition, the MCID may be useful to ECPs in evaluating contact lens performance and helping them interpret the clinical meaning or relevance of these subjective responses.

Various approaches can be considered as viable methods of determining MCID. As there is yet no “gold standard” methodology, current thinking suggests that the estimation of MCID should be based on multiple approaches with the aim of converging into a single MCID value or small range of values.^15^ The methods for determining MCID are mainly grouped into two categories: *anchor-based* and *distribution-based* approaches.

The *anchor-based* approach compares the difference in score on a VAS or other rating scale for an outcome of interest to an external measure, called an *anchor*. Anchors can be derived from clinical tests, expert opinion, or PROMs.^13^^,^^15^ An example of an *anchor* is a five-point Likert preference scale. In a contact lens context, a Likert scale might allow participants to indicate their preference between two study lenses with regard to an outcome of interest (e.g., comfort or dryness). Typically, the five response options would include “strong” or “slight” preference for either of the lenses and “no preference.” It is reasonable to assume that if a participant indicates a slight preference between two products, then this can be used to determine the MCID for that individual.^5^ The difference in performance is significant enough to elicit a slight preference, suggesting clinical relevance, yet not so substantial as to denote a strong preference. For anchor-based approaches, the accurate estimation of MCID is dependent on the association between the external anchor and the outcome of interest. It has been proposed that a correlation of at least 0.3 suggests validity of the anchor.^15^

*Distribution-based* methods have also been used to help determine MCID. These approaches rely on statistical measures of the distribution of the outcome of interest such as the standard deviation (SD), effect size or standard error of measurement.^16^ A commonly-used approach is to calculate a proportion of the population SD of the rating scale under study.^15^ For example, previous work has reported a tendency for the MCID to converge into the 0.5 SD criteria.^17^^,^^18^ Distribution-based methods offer a standardized, efficient, and simple approach for MCID estimation; however, these methods are not derived from individual patient perception and therefore may not directly reflect clinical meaning from the patient's perspective.^13^^,^^17^ It is therefore recommended that distribution-based methods be used as supportive information for MCID estimates from anchor-based approaches.^15^

The purpose of this study was to determine MCID for contact lens-related subjective responses and to explore whether MCID values differ between subjective responses and study designs by conducting a retrospective analysis of data from previous clinical studies.

## Methods

### Bilateral Studies

A retrospective analysis was conducted on seven one-week, dispensing clinical crossover studies conducted at The University of Manchester between 2013 and 2019. These studies met the following inclusion criteria: bilateral crossover design, use of 0–100 VAS ratings scales for ocular subjective responses, use of 5-Likert preference rating scale for the same subjective domains and commissioned and funded by CooperVision Inc. Five of these studies were randomized whereas two were nonrandomized studies. For each study, ethical approval was granted from the University Research Ethics Committee of The University of Manchester prior to participant recruitment. The studies conformed to the tenets of the Declaration of Helsinki and all participants provided written informed consent prior to enrolment. All participants had successfully worn soft contact lenses within the last six months and met the inclusion and exclusion criteria outlined in Table 1.

**Table 1.** Inclusion and Exclusion Criteria

Inclusion Criteria	Exclusion Criteria
Age ≥18 years	Ocular disorder or abnormality contraindicating contact lens wear
Current wearer of soft contact lenses (within the last 6 months)	Systemic disorder or infectious disease contraindicating contact lens wear
Satisfactory fitting with study lenses	Corneal distortion from previous hard or rigid lens wear or keratoconus
At least 0.20 logMAR distance high-contrast visual acuity in each eye with study lenses	Pregnant or breastfeeding Use of topical medication

As part of these bilateral studies, participants wore two different contact lenses for one week each in a “matched pair” manner (i.e., the lens in the right eye was the same lens type as the lens in the left eye). The contact lenses investigated were commercially available spherical, daily disposable soft contact lenses, including hydrogel and silicone hydrogel materials, with a range of powers of −0.25 D to −7.50 D. The contact lens foils were overlabeled so that participants remained masked to the identity of the study lenses. Each participant was required to wear the study lenses for a minimum of eight hours per day, five days per week, and to return for the one-week follow-up visit wearing the study lenses for at least two hours.

**Figure 2.**
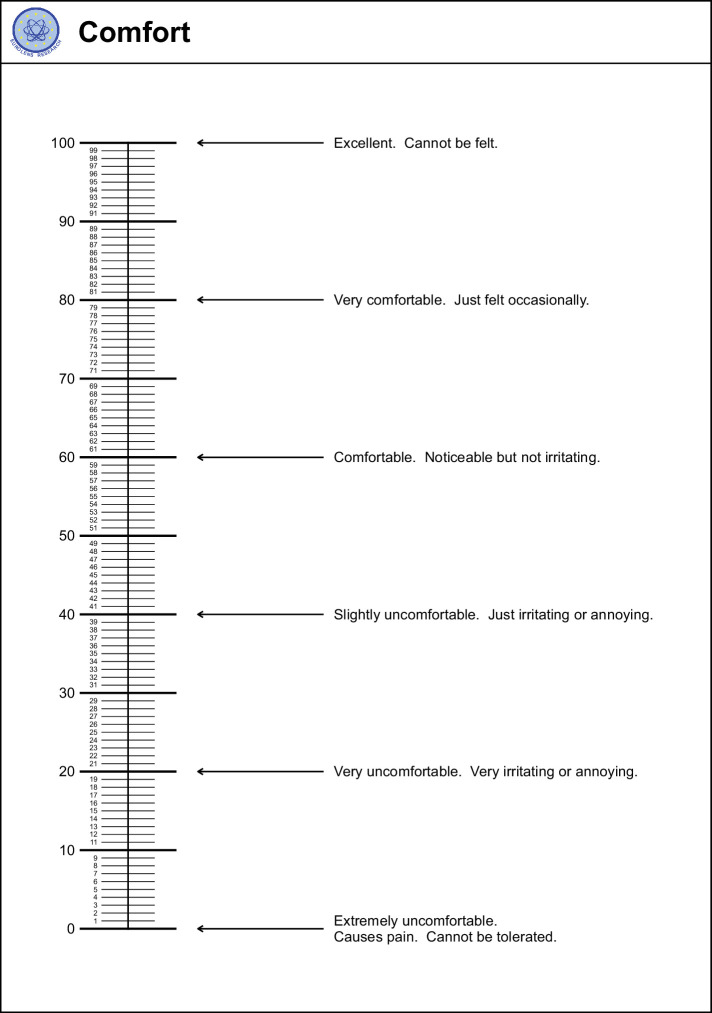
Annotated VAS for comfort used in the bilateral studies.

At the one-week follow-up visit, after having worn the lenses for at least two hours, participants were asked to rate comfort, dryness, vision, ease of insertion and/or ease of removal using 0–100 VAS with descriptors at 20-point intervals (Fig. 2 and Supplementary Figures). At the end of the second and final one-week follow-up visit, participants were asked to indicate their contact lens preference separately for comfort, dryness, vision and handling using a five-point preference scale, comprising strong or slight preference for either of the two lenses worn, or no preference. Data from these studies were pooled for the MCID estimation.

### Contralateral Studies

A retrospective analysis was performed on 14 one-day, nondispensing, contralateral studies undertaken between 2016 and 2021 at CooperVision Inc. These studies met the following inclusion criteria: contralateral design, use of 0–100 VAS ratings scales for ocular subjective responses, use of 5-Likert preference rating scales for the same subjective domains and commissioned and funded by CooperVision Inc. Ethical approval was granted from the University of California, Berkeley Committee for Protection of Human Subjects (nine studies), Sterling Institutional Review Board (two studies) and Indiana University Institutional Review Board (three studies) before participant recruitment. The studies conformed to the tenets of the Declaration of Helsinki, and all participants provided written informed consent before enrollment. All the participants met the inclusion and exclusion criteria outlined in Table 1.

**Figure 3.**
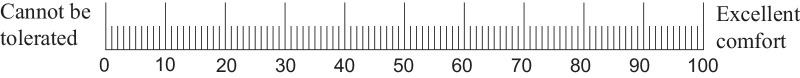
VAS for comfort used in the contralateral studies.

As part of these contralateral studies, participants wore an unmatched pair of two different soft contact lenses (i.e., the lens in the right eye was a different lens type to the lens in the left eye) for six hours. Comfort was assessed for each eye, using a 0–100 VAS with descriptors at 0 (cannot be tolerated) and 100 (excellent comfort) (Fig. 3), on insertion and after six hours of lens wear. At each timepoint, participants indicated their eye preference in terms of comfort using a five-point preference scale, comprising strong or slight preference for either eye or no preference. The contact lenses assessed were commercially available spherical soft contact lenses, including both daily disposable and reusable lenses, as well as hydrogel and silicone hydrogel materials, with a range of powers of −1.00 D to −6.00 D. Participants were masked to the identity of the study lenses. Data from these studies were combined for the MCID estimation.

### Statistical Analysis and MCID Determination

Statistical analysis was conducted in R Version 3.6.3^19^ with figures produced using the package ggplot2.^20^ The absolute mean difference in VAS scores between contact lenses was calculated for the “slight preference,” “strong preference,” and “no preference” categories for each variable of interest. Spearman correlation between the absolute mean difference in VAS score and the preference rating scale was calculated for each variable of interest. A Spearman correlation of 0.3 or greater was considered to show validity of the anchor for the estimation of MCID.^15^

Because no consensus has been reached regarding the optimal MCID calculation method, the following four estimates of the MCID (a combination of anchor-based and distribution-based approaches) were calculated, and furthermore the mean of the four MCID estimates was also calculated for each variable of interest separately. Because the rating scale scores are integer based, the MCID average was rounded to the nearest whole integer.

1. Anchor-based approach: The mean VAS score difference for all those indicating a slight preference (termed “mean difference”)^5^^,^^15^
2. Anchor-based approach: The lower limit of the 95% confidence interval (CI) VAS score difference for all those indicating a slight preference (termed “LL 95% CI difference”)
3. Anchor-based approach: The difference between the mean difference in VAS score for “slight preference” and the mean difference in VAS score for “no preference” (termed “category difference”)^15^
4. Distribution-based approach: Fifty percent of the standard deviation of the VAS scores at the first follow-up visit (bilateral studies) or for Lens 1 (contralateral studies) (termed “0.5 SD”)^17^

## Results

### Bilateral Studies

Across the seven bilateral studies, there were 326 participants (244 female; 82 male) with a mean age (±SD) of 29.1 ± 9.3 years and a range of 18 to 61 years.

Table 2 shows the results for the Spearman correlation between the absolute difference in VAS score (between contact lenses) and the preference ratings. The correlation was greater than the recommended threshold of 0.3 for comfort, dryness, vision, and ease of insertion, showing acceptable association between the VAS score difference and the preference rating scale. Given that the correlation between the handling preference rating scale and ease of removal VAS rating scale did not meet the 0.3 threshold, no further analysis was performed for this domain.

**Table 2.** Spearman Correlation Between the Absolute Difference in VAS Score Between Contact Lenses and the Preference Ratings—Bilateral Studies

	N	Spearman's ρ	*P* Value
Comfort	326	0.52	<0.0001
Dryness	307	0.49	<0.0001
Vision	307	0.58	<0.0001
Ease of insertion	173	0.50	<0.0001
Ease of removal	173	0.26	0.0007

**Figure 4.**
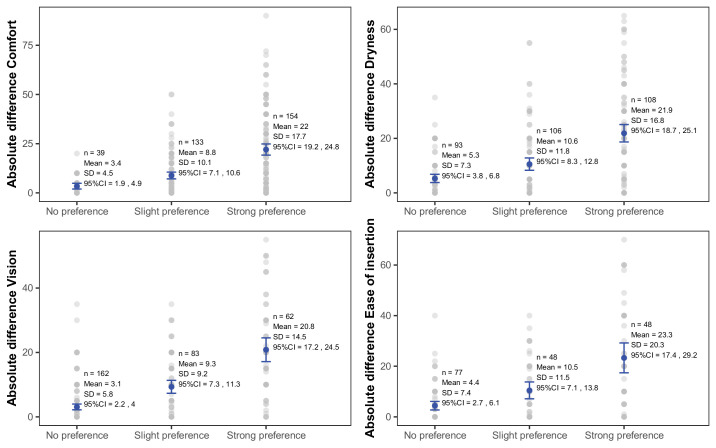
Absolute differences in VAS score between contact lenses according to the preference category for comfort, vision, dryness, and ease of insertion—bilateral studies

Figure 4 shows the differences in VAS score between contact lenses for each preference strength for the variables of interest. The MCID estimates from the four calculation methods for the various variables of interest are shown in Table 3. The four calculation methods generated a small range of MCID values. The averaged MCID was 7.2 (range 5.4–8.8) for comfort, 8.1 (5.2–10.6) for dryness, 7.1 (5.5–9.3) for vision, and 7.6 (6.0–10.5) for ease of insertion.

**Table 3.** The MCID Estimates for Comfort, Dryness, Vision, and Ease of Insertion—Bilateral Studies

MCID Calculation Method	Comfort	Dryness	Vision	Ease of Insertion
Mean difference	8.8	10.6	9.3	10.5
LL 95% CI difference	7.1	8.3	7.3	7.1
Category difference	5.4	5.2	6.2	6.0
1/2 SD	7.5	8.5	5.5	6.7
**Average**	**7.2 ≈ 7**	**8.1 ≈ 8**	**7.1 ≈ 7**	**7.6 ≈ 8**

Mean difference = the mean VAS score difference for all those indicating a slight preference; LL 95% CI difference = the lower limit of the 95% confidence interval VAS score difference for all those indicating a slight preference; category difference = the difference between the mean difference in VAS score for “slight preference” and the mean difference in VAS score for “no preference”; and 0.5 SD = 50% of the SD of the VAS scores at the first follow-up visit.

### Contralateral Studies

Across the 14 contralateral studies, there were 311 participants (237 female; 74 male) with a mean age (±SD) of 26.6 ± 6.6 years and a range of 18 to 60 years. The Spearman correlation between the absolute difference in VAS score (between contact lenses) and the preference rating scale was greater than 0.3, showing acceptable association for the MCID estimation (comfort at insertion ρ = 0.76, *P* < 0.0001; comfort at end of day ρ = 0.85, *P* < 0.0001).

**Figure 5.**
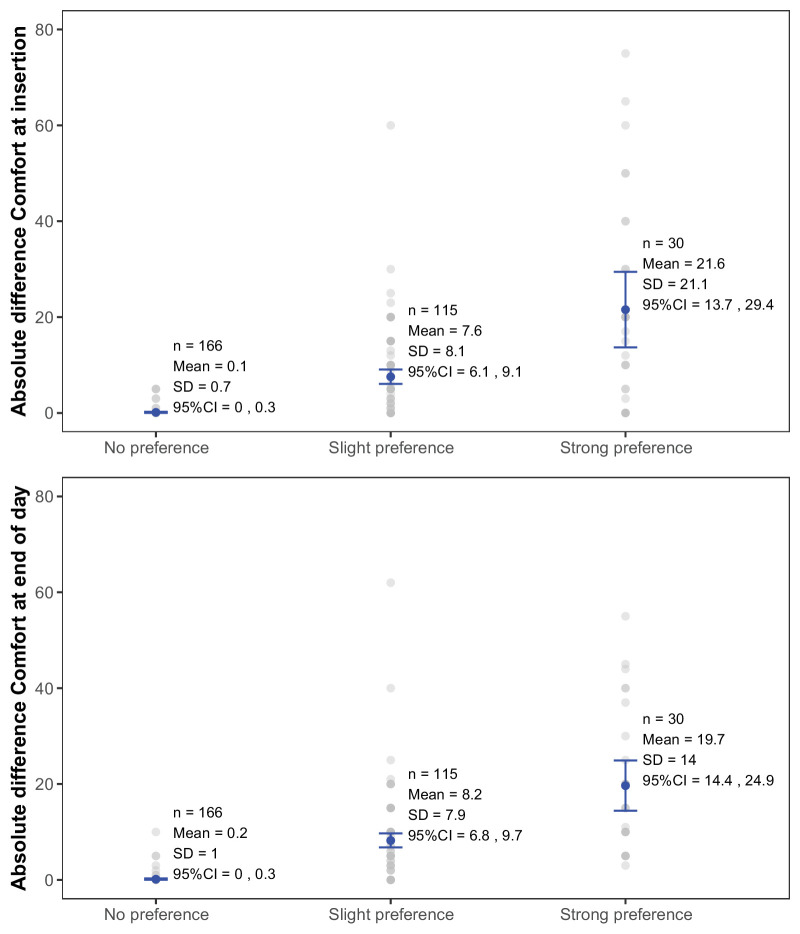
Absolute differences in VAS score between contact lenses according to the preference category for comfort at insertion and end of day—contralateral studies.

Figure 5 shows the differences in VAS score between contact lenses for each preference category for comfort at insertion and end of day. All the MCID estimates are shown in Table 4. The averaged MCID was 6.9 (range 6.1–7.6) for comfort at insertion and 7.5 (6.8–8.2) for end-of-day comfort.

**Table 4.** The MCID Estimates for Comfort at Insertion and End of Day—Contralateral Studies

MCID Calculation Method	Comfort at Insertion	Comfort at End of Day
Mean difference	7.6	8.2
LL 95% CI difference	6.1	6.8
Category difference	7.4	8.1
1/2 SD	6.4	6.9
**Average**	**6.9 ≈ 7**	**7.5 ≈ 8**

Mean difference = the mean VAS score difference for all those indicating a slight preference; LL 95% CI difference = the lower limit of the 95% CI VAS score difference for all those indicating a slight preference; category difference = the difference between the mean difference in VAS score for “slight preference” and the mean difference in VAS score for “no preference’; and 0.5 SD = 50% of the SD of the VAS scores for Lens 1.

According to Papas et al.,^5^ “hypo-raters” were defined as participants who reported a preference, either slight or strong, but had a zero VAS score difference between lenses and “hyper-raters” as participants who reported no preference but had a nonzero VAS score difference between lenses.^5^ Across the range of studies, some participants were classified as “hypo-raters” (range 0%–10% of total sample size) and “hyper-raters” (range 2%–20%). It was also observed that a small number of participants (range 0.3%–6%) indicated a preference for a contact lens but rated their preferred lens worse than the other lens. These participants are referred to as “contradictory raters.” Table 5 shows the number of participants who were classified as “hypo-raters,” “hyper-raters,” and “contradictory raters.” These phenomena were observed across all the subjective responses and appeared to be more frequent in those reporting a slight preference.

**Table 5.** Number of Participants (% of Sample Size of Each Variable) Classified as “Hypo-Raters,” “Hyper-Raters,” and “Contradictory Raters” Across the Seven Bilateral Studies and 14 Contralateral Studies

	Hypo-Raters/Slight Preference	Hypo-Raters/Strong Preference	Hyper-Raters	Contradictory Raters/Slight Preference	Contradictory Raters/Strong Preference
Bilateral					
Comfort	34 (10%)	8 (2%)	21 (6%)	18 (6%)	7 (2%)
Dryness	25 (8%)	8 (3%)	51 (17%)	19 (6%)	5 (2%)
Vision	23 (7%)	4 (1%)	62 (20%)	15 (5%)	1 (0.3%)
Ease of insertion	16 (9%)	6 (3%)	32 (18%)	9 (5%)	4 (2%)
Contralateral					
Comfort at insertion	25 (8%)	6 (2%)	7 (2%)	8 (3%)	1 (0.3%)
Comfort at end of day	18 (6%)	0 (0%)	7 (2%)	7 (2%)	3 (1%)

## Discussion

This work has demonstrated remarkably similar MCID values across a range of subjective variables; MCID values were on average seven units on a 0–100 VAS, essentially the same as the values of “7 to 8 units” cited by Papas and colleagues.^5^ No standardized methodology for determining MCID has been established, and thus multiple calculation techniques have been proposed in the literature. By definition, different MCID calculation approaches are likely to produce a variety of MCID values. The four MCID calculation methods used in this work resulted in a relatively small range of values. The largest MCID values were consistently derived from the “mean difference” approach, whereas the calculation method generating the smallest MCID value varied across the range of subjective variables. The study findings support the use of seven units as the MCID for subjective measures of comfort, vision, dryness, and ease of insertion on 0–100 scales. The observation that the values are near-identical despite the range of subjective experiences might indicate that this range could also be used for other contact lens-related (and, indeed, other ocular or general) measures of subjective experience.

Papas and colleagues^5^ estimated the JND for comfort ratings in subjects wearing an identical pair of contact lenses. Given that different contact lenses were worn in the present contralateral studies, a larger difference than the JND might have been expected. Instead, the MCID values calculated were very similar. The range of MCID values was very narrow for the contralateral studies, indeed narrower than that observed in the bilateral studies. The comparison of the MCID results between contralateral and bilateral studies in this study is limited by the fact that the VAS differed in design. Bilateral studies used a vertical scale with written descriptors at 20-point intervals whilst contralateral studies used a horizontal scale with written descriptors at 0 and 100. In addition, overall comfort was collected in the bilateral studies whereas insertion/end-of-day comfort was measured in the contralateral studies. Although it is generally suggested that MCID values only applied to the specific VAS used^13^ the results of the present work appear to challenge this claim. Rating scales of similar range (i.e., 0 to 100) but of different design produced very similar MCID values and suggest that small differences in this type of scale and study designs does not seem to significantly affect the MCID estimate of subjective contact lens-related comfort responses. The remarkably similar MCID obtained in this work despite the variety of subjective responses, rating scale designs and study designs could also reflect the limit of human discrimination ability.

Anchor-based approaches for estimating the MCID require a significant correlation between the external anchor and the outcome of interest, with a recommended correlation coefficient of 0.3 or greater.^15^ The moderate correlation observed between the difference in VAS score and the preference rating scale suggests the validity of the anchor for MCID estimation.^15^ Although generally good association was observed between these two outcomes, some values appeared to deviate, with some participants being classified as “hypo-raters,” “hyper-raters,” and “contradictory raters.” These phenomena may simply indicate the measurement error of the outcomes (e.g., the difficulty of accurately expressing human experiences on numeric scales, random error, etc.). It is also possible that a recall bias may have affected the responses of the preference rating scale in the bilateral studies. Participants rated their lens preference at the final follow-up, after two weeks of lens wear (i.e., one week on each lens type) and then needed to recall their experience with the first lens to compare it with the second lens to make a judgment of the extent of the difference between lenses and state their preference accordingly. It is possible that the ability of the participant to retain this memory, as well as the influence of the current status may have affected their responses. This seems to be supported by the fact that these phenomena, particularly the “hyper-rating,” were less frequently observed in the contralateral studies, where participants were able to make a direct real-time comparison of contact lenses.

Unfortunately, no repeated measures were captured in these studies, making it challenging to determine whether these phenomena were just random or consistent among participants. Nevertheless, it was observed that the majority of these participants exhibited one or more of these abnormal behaviors occasionally but never consistently across all symptoms evaluated. Excluding these phenomena from the analysis, which involves omitting non-zero values in the “no preference” category and zero values in the “slight preference” category, unsurprisingly leads to larger MCID values. However, excluding these phenomena from the estimation of the MCID is hard to justify, because these data points represent genuine responses from participants and are therefore included in the estimation of MCID values.

For lens handling, two VAS scales had been used in these studies, termed “ease of insertion” and “ease of removal,” whereas the item assessed by the preference-rating scale was simply termed “handling.” Although ease of insertion VAS data showed acceptable correlation with the handling preference scale, this was not the case for ease of removal, which did not reach the pre-established threshold, and therefore the MCID estimation was not possible for this domain. This finding could suggest that lens preference in terms of overall handling is more influenced by the ease of lens insertion than the ease of lens removal. Further research could explore which aspects of lens handling drive a preference for a contact lens, as well as attempting to determine the MCID for ease of lens removal by using a preference scale specifically for this item. This information would be particularly useful in new wearers as handling has been reported to be a key reason for discontinuing contact lens wear in the early weeks.^21^

It is feasible that MCID may vary across the range of the VAS scale.^22^ The contact lenses investigated in this study were all commercially available products, which generally performed well, with scores more commonly observed in the upper half of the scale. Consequently, further exploration of this hypothesis was not possible. The contact lenses used in this study were all spherical soft contact lenses representative of those worn in the real world. The influence of lens characteristics such as toric or multifocal designs on the MCID should be explored in future investigations.

The study population consisted of successful CL wearers, most of whom were female and aged under 40 years; therefore the results of this work are applicable to this specific demographic, which is representative of typical contact lens wearers. This relatively unbalanced cohort in terms of age and sex limits the ability of this study to reliably determine the influence of these demographics on MCID values. Future research is needed to ascertain whether MCID values vary with individual demographic factors such as age, sex, or ethnicity. Additionally, since MCID can vary across clinical contexts, future studies should be conducted to determine if MCID values differ among specific populations, such as symptomatic contact lens wearers, new wearers/neophytes or children. Until further evidence is provided, the use of seven units on a 0–100 rating scale seems reasonable.

MCID values can also be used as the *equivalence* or *non-inferiority* margin when planning clinical studies. An important consideration is that the sample size required for clinical studies depends directly on the size of the *equivalence/non-inferiority* margin. As a general rule, the smaller the margin, the larger the sample size required for *equivalence* and *non-inferiority* testing.^23^^,^^24^ The sample size required also depends in part on the anticipated difference in score between the contact lenses investigated. In general, the closer the anticipated difference is to the *equivalence/non-inferiority* margin, the larger the sample size required to claim *equivalence* or *non-inferiority.* The sample size required for *non-inferiority* is generally slightly smaller than that required for *equivalence*.^23^^,^^24^ If information about the expected relative performance of two contact lenses is known, it can be used to provide for a more accurate power assessment. If nothing is known about the likely relative performance of two products (e.g., an existing contact lens vs. a very new contact lens), anticipating a difference of zero would seem appropriate.

There is no standard methodology for estimating MCID, and several approaches have been described in the literature. The methodology used in the present study aligns with recommendations from previous publications.^15^^,^^25^ One strength of this approach is the selected anchor—the preference rating scale—which relies on the participants’ perspective, reflects a small but important difference, is easily understandable, and shows a moderate correlation with the PROMs (i.e., VAS). Both the anchor and PROMs measure the same or closely related constructs under a relatively short recall period—two weeks for the bilateral studies and six hours for the contralateral studies. Considering that MCID is dependent on a range of factors, a combination of distribution-based and anchor-based MCID estimates was used, as well as analytical methods to account for this diversity, using triangulation to converge into a single or small range of MCID values.

However, there are certain limitations in this retrospective work, which is somewhat restricted by the available data. Although the use of multiple anchors has been recommended, only a single anchor—the preference rating scale—was used for the estimation of the MCID. Additionally, the assessment of lens preference was undertaken on a single occasion, so intraperson consistency could not be determined. Future research should explore the validity of these findings under different contexts such as varying participant and lens characteristics, as well as using multiple anchors for MCID estimation.

## Conclusions

This work has demonstrated very similar MCID values, seven units on a 0–100 VAS, across a range of ocular, contact lens–related subjective responses including comfort, dryness, vision, and ease of insertion, in a population of habitual soft contact lens wearers, most of whom were female and aged under 40 years. Very similar MCID values were also obtained for ratings of comfort for both bilateral and contralateral study designs. This information may help in the design and statistical analysis of clinical studies, as well as in the interpretation of these subjective responses.

## Supplementary Material

Supplement 1
