# Supplementary figures and images for: Determination of the Minimal Clinically Important Difference (MCID) for Ocular Subjective Responses
Source: Transl Vis Sci Technol. 2024 Aug 16;13(8):28. doi: 10.1167/tvst.13.8.28 (PMC11343006; doi:10.1167/tvst.13.8.28)

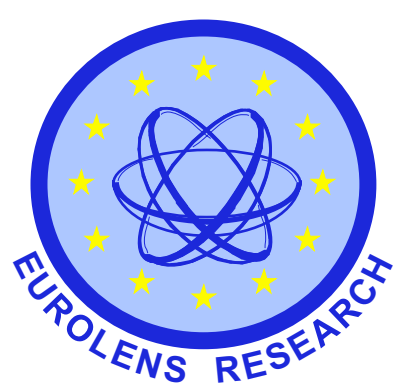

# Vision

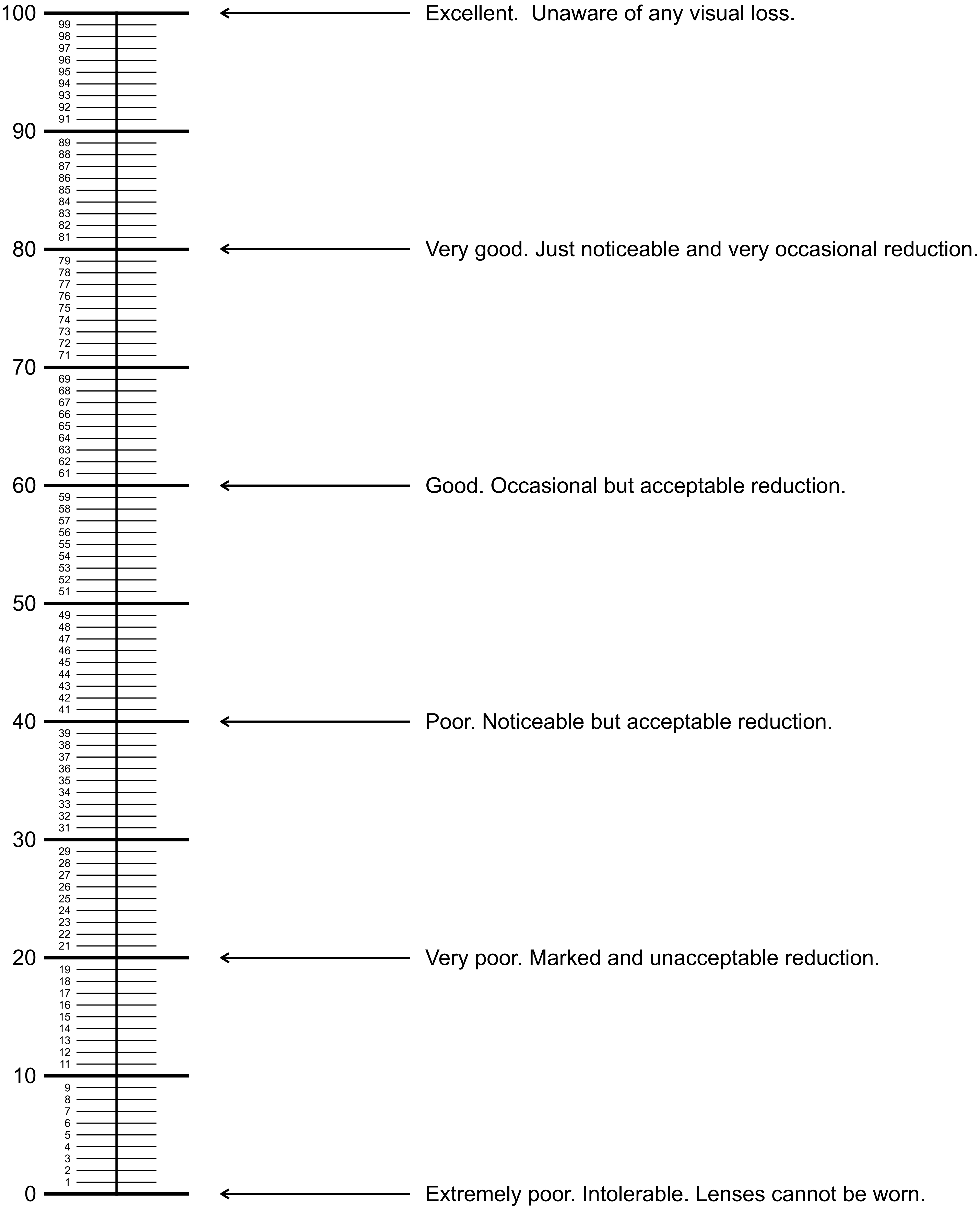

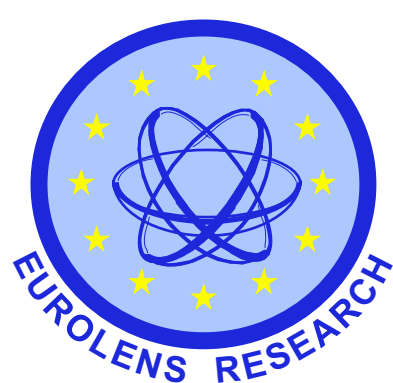

# Ease of lens insertion

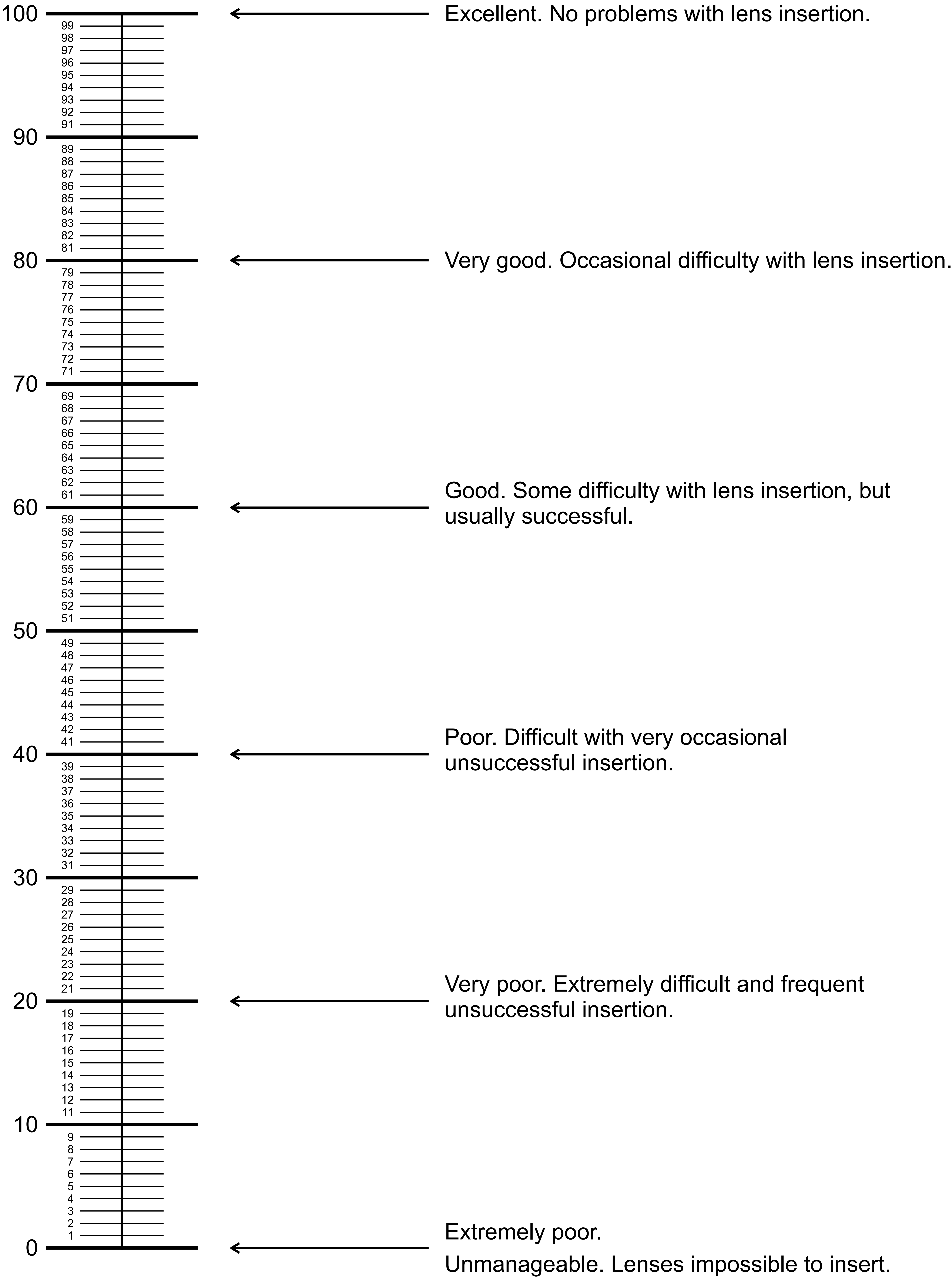

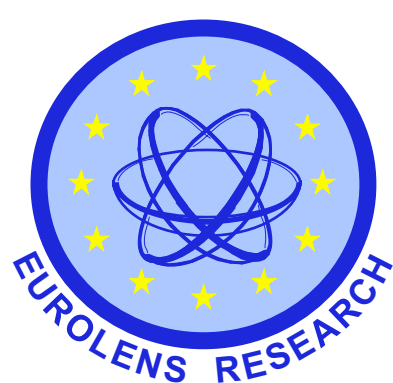

# Ease of lens removal

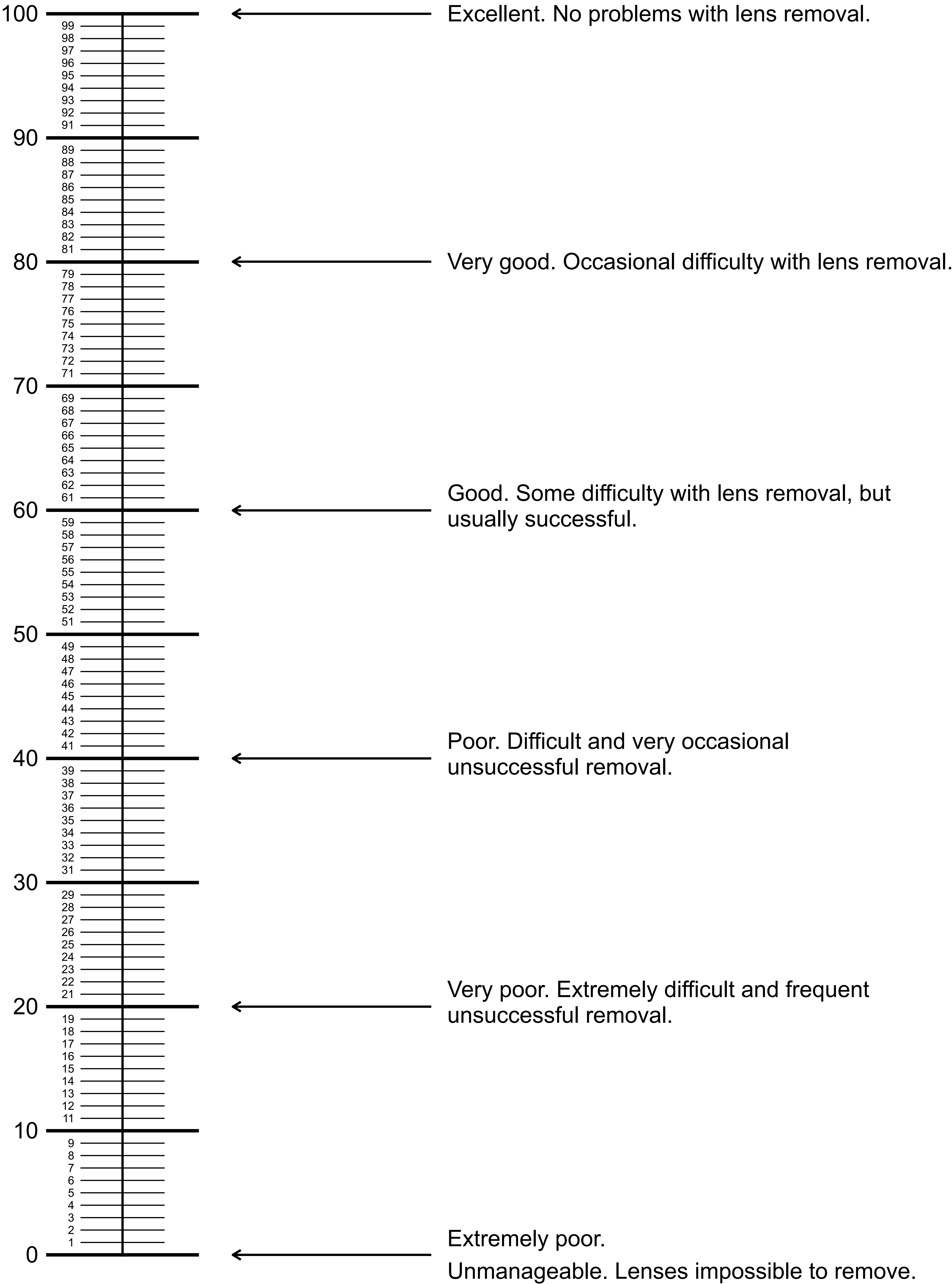

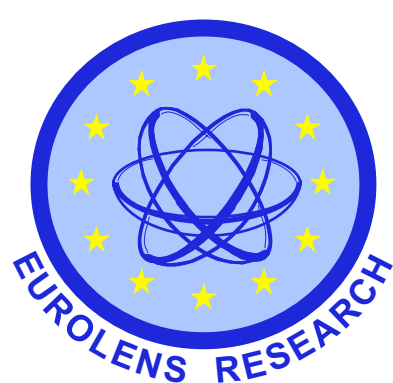

# Dryness

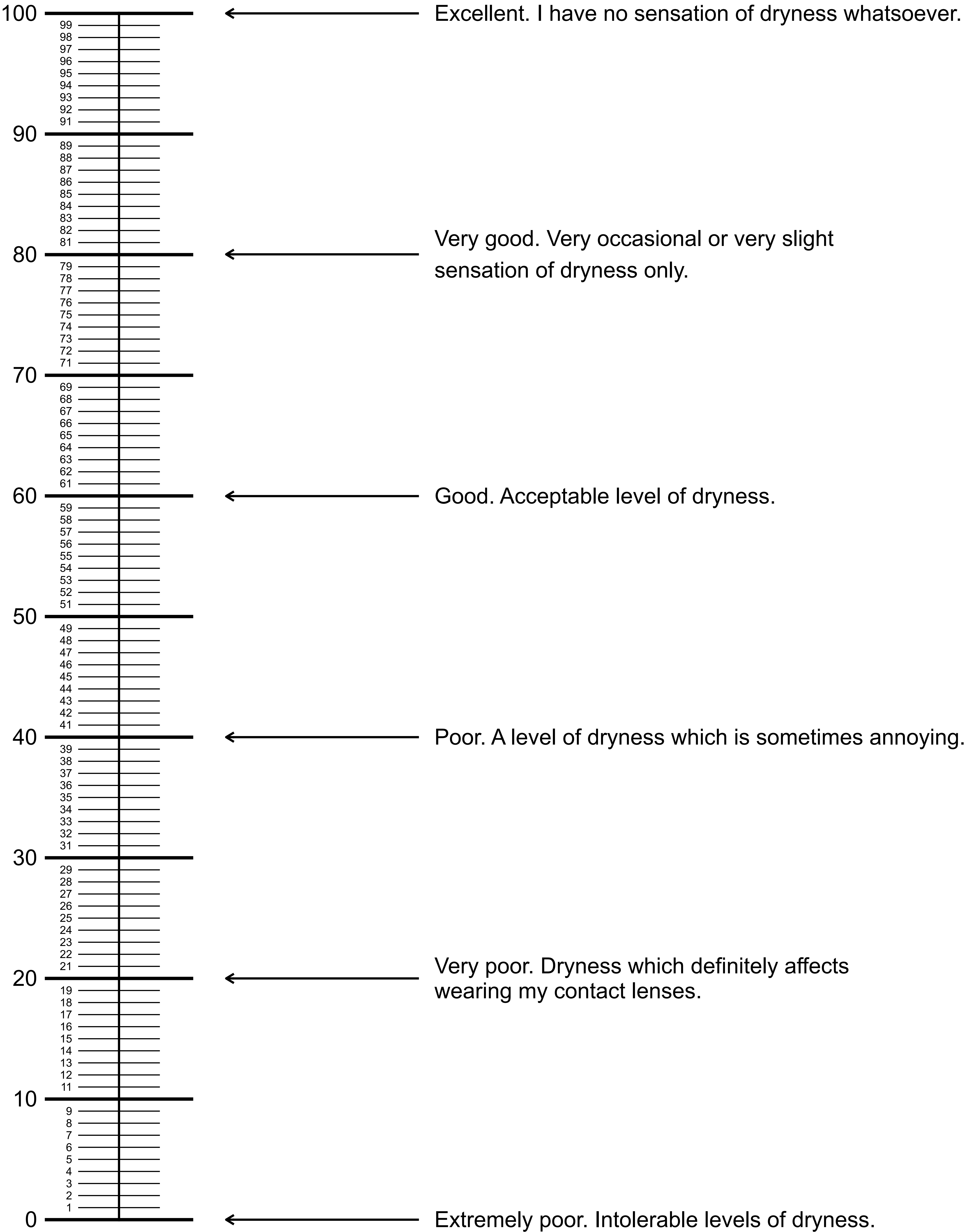

Supplement: Supplement 1 [file tvst-13-8-28_s001.pdf]
